# Molecular Identification and mRNA Expression Profiles of Galectin-9 Gene in Red Sea Bream (*Pagrus major*) Infected with Pathogens

**DOI:** 10.3390/ani11010139

**Published:** 2021-01-11

**Authors:** Kwang-Min Choi, Min-Soo Joo, Dong-Hee Cho, Won-Sik Woo, Gyoungsik Kang, Min Jin Heo, Do-Hyung Kim, Chan-Il Park

**Affiliations:** 1 Department of Marine Biology & Aquaculture, College of Marine Science, Gyeongsang National University, 455, Tongyeong 650-160, Korea; tricolo1229@naver.com (K.-M.C.); fkffkxodn@hanmail.net (M.-S.J.); nhjhyh@hanmail.net (D.-H.C.); dnstory@hanmail.net (W.-S.W.); gyoungsikkang@gmail.com (G.K.); benny0911@naver.com (M.J.H.); 2 Department of Aquatic Life Medicine, College of Fisheries Science, Pukyong National University, 45, Yongso-ro, Nam-Gu., Busan 48513, Korea; dhkim@pknu.ac.kr

**Keywords:** aquaculture, gene profiling, *Streptococcus iniae*, *Edwardsiella piscicida*, iridovirus

## Abstract

**Simple Summary:**

We identified the sequence encoding galectin-9 from *Pagrus major* and subsequently investigated the molecular characteristics and changes in gene expression patterns in response to artificial infection with major pathogens. Overall, our data suggest that galectin-9 plays a pivotal role in the immune system of *P. major*. The findings of this study can potentially serve as a reference for understanding the function of galectin-9 in the *P. major* immune system; moreover, galectin-9 has been identified as a potential candidate for use as a disease-related molecular marker.

**Abstract:**

Galectin (*Gal*) is a member of a family of β-galactoside-binding lectin. The members of this family play important roles in the recognition of carbohydrate ligands and in various other biological processes. In this study, we identified the gene encoding *Gal-9* in *Pagrus major* (*PmGal-9*) and analyzed its expression in various tissues after pathogen challenge. Alignment analysis revealed that the two galactose-binding lectin domains of the deduced protein were highly conserved among all the teleosts. Phylogenetic analysis revealed that *PmGal-9* is most closely related to the *Gal-9* gene of gilthead sea bream. *PmGal-9* was ubiquitously expressed in all tissues analyzed but was predominantly expressed in the spleen, head kidney, and intestine. After challenges with major microbial pathogens (*Edwardsiella piscicida*, *Streptococcus iniae*, or red sea bream iridovirus) of red sea bream, *PmGal-9* mRNA expression was significantly regulated in most immune-related tissues. These results suggested that *PmGal-9* not only plays an important role in the immune system of red sea bream but is also a possible inflammatory marker for pathogenic diseases.

## 1. Introduction

Galectins (*Gals*) are a family of β-galactoside-binding lectin. They were formerly known as S-type or S-lac lectins [1,2]. *Gals* play significant roles during innate immune responses by mediating the recognition of pathogens by host cells and are involved in the elimination of pathogens; they interact with carbohydrates on the surface of invading microorganisms and function as pattern recognition receptors (PRRs) in both vertebrates and invertebrates [3]. In addition, they are involved in many homeostasis and physiological processes such as apoptosis, inflammation, immune responses, cell migration, proliferation, adhesion, autophagy, and signal transduction [4,5,6]. *Gals* can be classified into three main types: the proto type (*Gal-1*, *-2*, *-5*, *-7*, *-10*, *-11*, *-13*, *-14*, *-15*, and *-16*), the tandem-repeat type (*Gal-4*, *-6*, *-8*, *-9*, and *-12*), and the chimera type (*Gal-3*), but they all have a highly conserved structure [7].

*Gal-9* was first isolated from mouse embryonic kidneys in 1997, demonstrating the family’s characteristically conserved sequence motifs and specific binding activity [8]. *Gal-9* is a ligand for the T cell immunoglobulin domain and mucin domain protein 3 and is involved in autoimmune- and allograft-related apoptosis actions [9,10]. In addition, previous studies confirmed the function of *Gal-9* in human plasmacytoid dendritic cells and B cells as well as its role in regulating TLR7/TLR9 signaling and its function as an eosinophil chemotactic factor [11]. Interferon-gamma-induced production of *Gal-9* has been shown to play an important role in the immune response by regulating the interaction between vascular walls and eosinophils, and researchers have reported the importance of *Gal-9* as an inflammatory mediator in acute dengue virus infection [12,13]. *Gal-9* induced the expression of apolipoprotein B editing complex 3 protein and mutated human immunodeficiency virus (HIV), weakening its infectivity and triggering the expression of various other anti-HIV factors [14].

Recently, there has been much interest in the correlation between *Gal-9* and tumors in humans, and many studies have reported its role as a prognostic factor [12,15,16]. In teleosts, *Gal-9* has been subjected to gene expression profiling and evaluations of its immunological function in a variety of species, but this research is still limited and has not been previously reported in red sea bream [17,18,19,20,21,22].

Red sea bream (*Pagrus major*) has been an important aquaculture species for many years in Korea and Japan. Although its production has increased due to the development of aquaculture technology, the disease is still a problem, especially those caused by dangerous pathogens that can lead to mass mortality, such as edwardsiellosis, streptococcosis, and red sea bream iridovirus (RSIV) disease [23]. Therefore, in order to solve problems related to pathogenic diseases, an understanding of the immune system and related research is necessary.

In this study, we obtained the cDNA sequence of *Gal-9* from *P. major* (*PmGal-9*) and identified its molecular biological characteristics using its deduced amino acid sequence. In addition, the expression pattern of *PmGal-9* mRNA was confirmed in the healthy state and after artificial infection by each pathogen.

## 2. Materials and Methods

### 2.1. Identification and Characterization of the Galectin-9 Gene

The coding sequence (CDS) of *PmGal-9* was obtained by next-generation sequencing (NGS) analysis in our previous study [24]. Suitability of the designed polymerase chain reaction (PCR) primers (forward: ATGGCTTTTAATCAGCAGTC, reverse: CTACACCACCACAGATGTCA) was confirmed using the primer3 primer-design program of the GENETYX software version 8.0 (GENETYX Corporation, Tokyo, Japan). Amplification of *PmGal-9* proceeded using ExPrime™ Tag Premix (GeNet Bio, Daejeon, Korea) according to the manufacturer’s instructions. The PCR product was then cloned into a pGEM-T Easy Vector (Promega, Madison, WI, USA), which was transformed into *Escherichia coli* (*E. coli*) JM109 competent cells. Plasmids were extracted using an Exprep Plasmid SV mini kit (GeneAll, Seoul, Korea). We confirmed the integrity of the sequence additional Sanger sequencing and predicted the amino acid sequence by using the GENETYX software version 8.0. *Gal-9* related amino acid sequences were retrieved from the National Biotechnology Information Center (http://www.ncbi.nlm.nih.gov/blast) database and used for multiple alignment analysis and phylogenetic analysis. Multiple alignment analysis of *PmGal-9* with deduced amino acid sequences of other species was performed with the ClustalX 2.1 program, and the characteristic domains and sequences of *Gal-9* were identified with the Expert Protein Analysis System PROSITE Scan tool (http://prosite.expasy.org) and simple modular architecture research tool (http://smart.embl-heidelberg.de/). For phylogenetic analysis, a tree was created using the MEGA 6.0 program (http://www.megasoftware.net) with a neighbor-joining algorithm. Support for each node was derived from 2000 resamplings.

### 2.2. Experimental Fish and Microorganisms

The red sea breams (weight: 173.2 ± 31.1 g, full length: 22.4 ± 0.9 cm) were provided by the Gyeongsangnam-do Fisheries Resources Research Institute and acclimated for two weeks in the laboratory. A total of 63 fish (21 individuals per group) were used to measure *PmGal-9* mRNA expression after pathogen challenge. The fish were randomly sampled prior to use in the experiment to identify major pathogen infections. All animal experiments were performed according to the guidelines of the Animal Protection and Use Committee of Gyeongsang National University (Approval Number: 2020-0002).

Bacteria and virus strain used in the experiments to analyze transcript expression levels after pathogen infection were obtained from the Fish Pathology Division of the National Institute of Fisheries Science (NIFS, Korea). The strains used were *Streptococcus iniae* (*S. iniae*) FP5228, *Edwardsiella piscicida* (*E. piscicida*) FSW910410, and RSIV.

### 2.3. Tissue Collection and Processing

For the analysis of *PmGal-9* mRNA transcript levels under healthy conditions, tissues from three independent fish were sampled after euthanasia via benzocaine (Sigma-Aldrich, St. Louis, MI, USA) and stored at −80 °C until total RNA extraction. Total RNA was extracted from the prepared tissues (brain, gills, head kidney, heart, intestine, liver, muscle, skin, spleen, stomach, and trunk kidney) according to the manufacturer’s instructions with RNAiso Plus reagent (Takara, Tokyo, Japan), and genomic DNA was removed. The extracted total RNA was measured for concentration and purity using a NanoVue spectrophotometer (GE Healthcare, London, UK) and synthesized into cDNA using a PrimeScript™ 1st strand cDNA Synthesis Kit (Takara, Tokyo, Japan) according to the manufacturer’s instructions. The PCR products were detected by electrophoresis on ethidium-bromide-stained agarose gels and visualized under ultraviolet light.

To measure the expression changes in *PmGal-9* mRNA after artificial infection by major pathogens in the red sea bream aquaculture industry, the experimental fish were divided into three groups, and *S. iniae* (1 × 10^5^ CFU/fish), *E. piscicida* (1 × 10^5^ CFU/fish) or RSIV (1 × 10^6^ copies/fish) were injected into their abdominal cavity. The control group was intraperitoneally injected with the same volume of phosphate-buffered saline (PBS) buffer. Then, the major tissues were aseptically extracted at various sampling times (1, 12, 24, 72, 120, and 168 h), and total RNA was extracted and synthesized into cDNA as in the method described above.

### 2.4. Quantitative Real-Time PCR

The expression level of the *PmGal-9* transcript was measured by quantitative real-time PCR using the prepared cDNA samples and specific primer sets (forward: CAGGCAGAATGCAGACATTG and reverse: GCTGAAGGTAGAGCCAGCAG). The SYBR green method was used, and real-time PCR was performed with TB Green™ Premix Ex Taq™ (Takara, Tokyo, Japan) and Thermal Cycler Dice Real Time System III (Takara, Tokyo, Japan) according to the manufacturer’s instructions. The measured threshold cycle (Ct) values were calculated using the Ct values of the red sea bream elongation factor 1-alpha gene (forward: CCTTCAAGTACGCCTGGGTG and reverse: CTGTGTCCAGGGGCATCAAT) and the delta-delta Ct method as the fold change relative to the control group. All experiments were repeated three times, and statistical analysis was performed using one-way analysis of variance (ANOVA) followed by Tukey’s multiple comparison test. The analysis was conducted using SPSS software version 19 (IBM, Chicago, IL, USA). Significance was indicated by the *p*-value (* *p* < 0.05 and ** *p* < 0.01).

## 3. Results

### 3.1. Identification and Characterization of PmGal-9 Sequence

**Figure 1:**
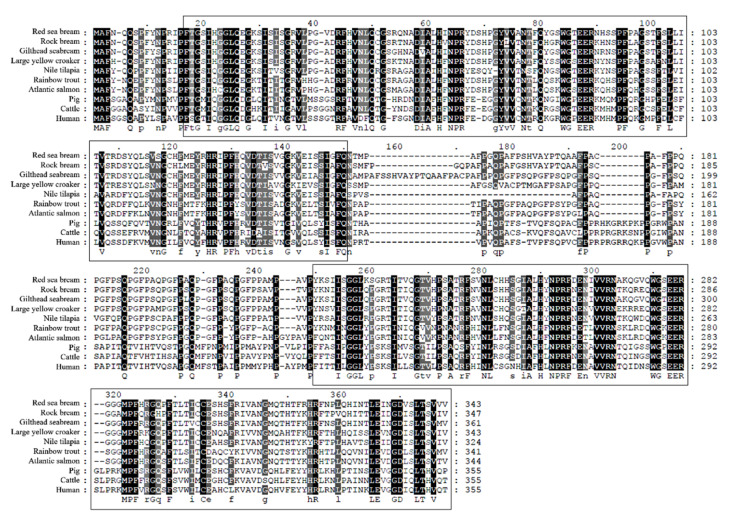
Multiple sequence alignment analysis of the deduced amino acid sequence of *PmGal-9* with fish *Gal-9* sequences. This analysis is based on the following sequence data: rock bream (ANN46244), gilthead sea bream (XP_030261557), large yellow croaker (XP_010754381), Nile tilapia (XP_003458375), rainbow trout (ACO08221), Atlantic salmon (ACI67584), pig (NP_999097), cattle (NP_001034266), and human (CAB93851). The predicted galactose-binding lectin domains are indicated by the box. Black boxes: identity = 100%; gray boxes: 80% ≤ identity < 100%; light gray boxes: 50% ≤ identity < 80%.

**Figure 2:**
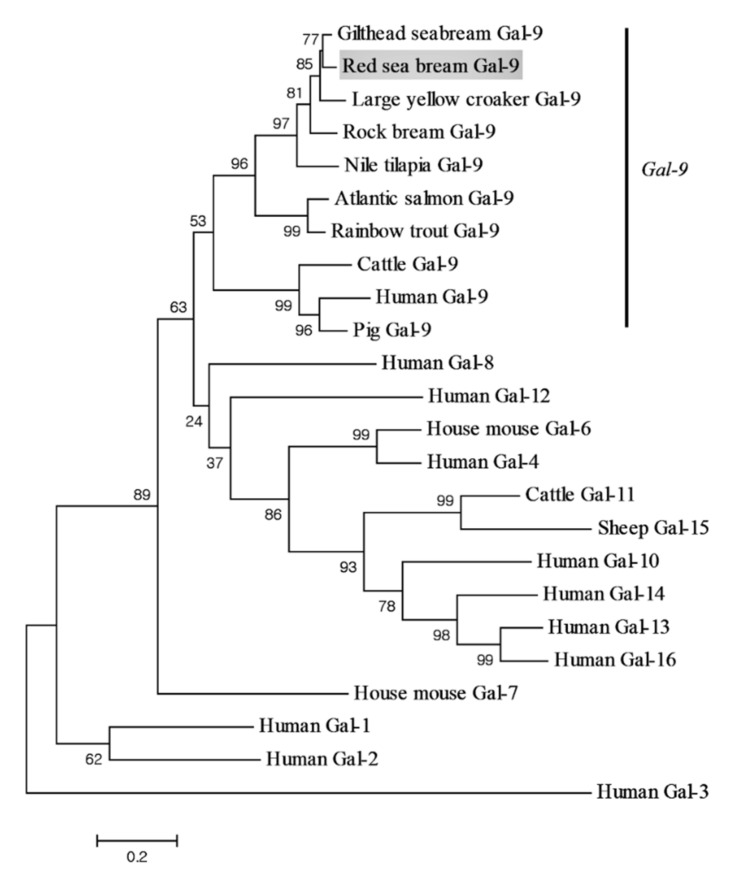
A phylogenetic tree of *PmGal-9* and other known *Gal-9* homologs based on the neighbor-joining (NJ) method. The scale bar indicates a branch length of 0.2. Numbers are bootstrap percentiles from 1000 replicates. This analysis is based on the following sequence data: Human *Gal-1* (NP_002296), Human *Gal-2* (NP_006489), Human *Gal-3* (BAA22164), Human *Gal-4* (NP_006140), House mouse *Gal-6* (AAI60275), House mouse *Gal-7* (NP_032522), Human *Gal-8* (AAF19370), Gilthead sea bream *Gal-9* (XP_030261557), Large yellow croaker *Gal-9* (XP_010754381), Rock bream *Gal-9* (ANN46244), Nile tilapia *Gal-9* (XP_003458375), Atlantic salmon *Gal-9* (ACI67584), Rainbow trout *Gal-9* (ACO08221), Cattle *Gal-9* (NP_001034266), Human *Gal-9* (CAB93851), Pig *Gal-9* (NP_999097), Human *Gal-10* (NP_001819), Cattle *Gal-11* (CBX54571), Human *Gal-12* (NP_001136007), Human *Gal-13* (ACR09640), Human *Gal-14* (ACR09644), Sheep *Gal-15* (NP_001009238), and Human *Gal-16* (NP_001177370).

The CDS of *PmGal-9* is 1032 bp long (GenBank accession No. QLI33832), which encodes a mature peptide of deduced 343 amino acids (aa) with a calculated molecular weight of 37.43 kDa and a theoretical isoelectric point of 9.11. *PmGal-9* has two putative galactose-binding lectin domains located at 16 to 148 and 217 to 343 aa (Figure 1). The deduced amino acid sequence alignment of the *PmGal-9* sequence with those from other species indicated their high shared identity (Table 1). Multiple sequence alignment showed that *PmGal-9* had the highest similarity to rock bream *Gal-9* (85.6%), followed by gilthead sea bream *Gal-9* (85.0%), large yellow croaker *Gal-9* (79.0%), and Nile tilapia *Gal-9* (72.9%). There was a comparatively low level of identity with pig *Gal-9* (45.4%), cattle *Gal-9* (44.0%), and human *Gal-9* (43.4%). Furthermore, a phylogenetic tree was constructed using the deduced amino acid sequences (Figure 2). *PmGal-9* formed distinct clusters with the *Gal-9* of teleosts, implying a closer relationship between *PmGal-9* and that of other teleosts.

### 3.2. Expression of PmGal-9 mRNA in Various Tissues

**Figure 3:**
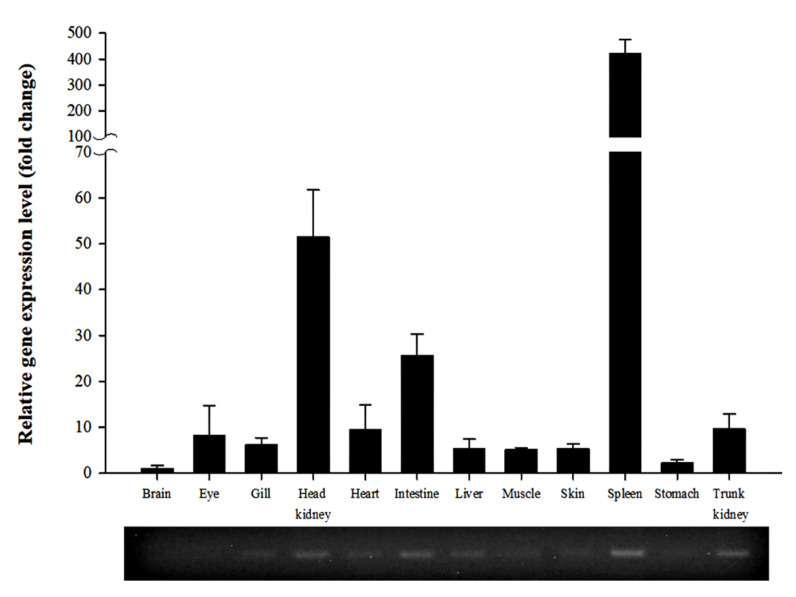
Expression level of *PmGal-9* mRNA in various tissues of healthy red sea bream. The *PmEF-1α* gene was used to normalize the RT-qPCR results. The expression level is expressed as the fold change compared to the expression level of *PmGal-9* mRNA in the brain. An agarose gel image of the PCR products is shown at the bottom. All data are presented as the mean ± SD from three independent cDNA samples with three replicates per sample.

We used RT-qPCR to extract total RNA from three healthy fish to investigate the distribution of *PmGal-9* mRNA in various tissues (Figure 3). It was expressed ubiquitously in all tissues (trunk kidney, head kidney, gill, spleen, heart, liver, brain, stomach, intestine, skin, muscle, and peripheral blood lymphocytes) analyzed. Compared with the brain, the spleen exhibited higher expression of *PmGal-9* (422.7-fold), followed by the head kidney (51.5-fold) and intestine (25.6-fold). In contrast, relatively low levels of expression were observed in the liver, muscle, skin, and stomach.

### 3.3. Expression of PmGal-9 mRNA after Pathogen Infection

**Figure 4:**
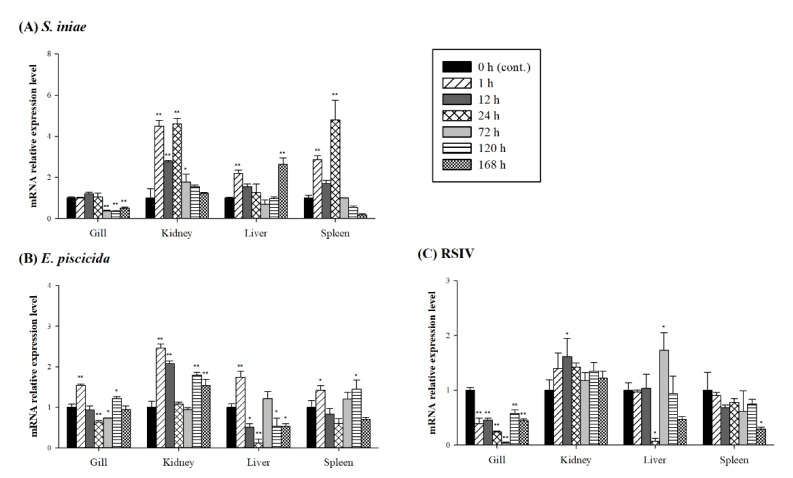
Expression level of *PmGal-9* mRNA in the gill, kidney, liver, and spleen of red sea bream after infection with (**A**): *Streptococcus iniae*, (**B**): *Edwardsiella piscicida*, or (**C**): red sea bream iridovirus (RSIV). Gene expression levels and their significance are represented as the mean ± SD. Asterisks indicate significant differences (* *p* < 0.05, ** *p* < 0.01) versus the control group.

The mRNA expression levels of *PmGal-9* in the gill, whole kidney, liver, and spleen at 1, 12, 24, 72, 120, and 168 h after pathogen challenge (*S. iniae*, *E. piscicida* or RSIV) were determined using RT-qPCR (Figure 4). The *PmGal-9* transcript levels were significantly up- or downregulated in all tested tissues and were increased more in response to bacterial infection than in response to viral infection. After *S. iniae* inoculation and during the initial stage of this artificial infection (between 1 and 24 h), *PmGal-9* mRNA transcript levels were highest in the gills, kidneys, and spleen and gradually declined thereafter. In contrast, although hepatic *PmGal-9* mRNA transcript levels were significantly upregulated at 1 h postinfection, the highest levels were achieved at 168 h, after a transient intervening decrease. After artificial *E. piscicida* infection, all examined tissues exhibited the highest *PmGal-9* mRNA transcript levels at 1 h, followed by a transient decline before re-upregulation at 72 and 120 h. After RSIV infection, gill *PmGal-9* mRNA transcript levels remained significantly downregulated for the duration of the infection period, and they were maintained in the spleen until 168 h, when significant downregulation became apparent. In the kidney, levels were significantly upregulated at 12 h postinfection, and then declined to baseline, where they remained for the duration of the infection period. Hepatic levels were significantly downregulated at 24 h postinfection, prior to significantly increasing at 72 h and then declining again.

## 4. Discussion

*Gal-9* forms a dimeric structure with a linker peptide bond, and a specific region at the N-terminus is known to be highly conserved between the *Gal* family [25,26,27]. Moreover, the structural features of *PmGal-9* were consistent with our results, as observed in previous reports where *Gal-9* was highly conserved throughout the teleost *Gal-9* sequence [19,22]; however, certain sequences in the galactose-binding lectin domains were missing compared to those in mammalian *Gal-9*, and in particular, the sequence of the linker peptide (151 to 249 aa) connecting the two galactose-binding lectin domains was confirmed to vary distinctly between species (Figure 1). In a study by Nishi et al., the lack of a linker peptide in *Gal-9* indicated more stability against proteolysis; however, some sequence mutations occurring in the galactose-binding lectin domain and changes in the sugar-binding activity were not confirmed [28]. Similarly, these were also reported in *Gal-7*, demonstrating that the mutation was not related to the ability of the protein to regulate apoptosis; instead, the activation of the galactose-binding lectin domain was required to inhibit the invasiveness of cancer cells [29]. On the other hand, compared to the wild type, the mutated *Gal-9* protein found in the canine gastrointestinal nematode parasite considerably decreased its affinity for carbohydrates or lost its activity [30]. Although the obtained results differ from those reported for the *Gal* homolog and are in conflict with the study purpose, they suggest the importance of the conservation of major residues—such as polar amino acids—within the galactose-binding lectin domain.

*Gal-9* is highly expressed in many immune organs, including the kidney, bone marrow, lymph nodes, spleen, and thymus. It is known to have a direct role in immune-related cells and systems [31,32,33,34]. In mammals, *Gal-9* interacts with the T cell immunoglobulin and mucin domain-containing protein 3 (TIM-3), a marker of various immune cells, and is involved in the apoptosis of T cells and the activation of dendritic cells [9,35,36]. In healthy *Pelteobagrus fulvidraco* and *Labeo rohita*, *PfGal-9* and *LrGal-9* mRNAs are most abundantly distributed in the blood, spleen, kidney, or intestine and least abundantly distributed in the muscle, skin, or brain [19,21]. In healthy rainbow trout, abundant expression was also found in lymphoid organs such as the thymus, head kidney, and spleen, all of which contain macrophages and lymphocytes [37]. On the other hand, different results have been reported for the fish species *Rhodeus uyekii*, *Larimichthys crocea*, and *Oreochromis niloticus*, and the differences in expression patterns were confirmed [18,20,22]. However, overall, the results clearly indicate that *Gal-9* mRNA expression is relatively abundant in major immune and peripheral lymphoid tissues in fish, which may be related to its previously reported immunological correlation with lymphoid cells. In addition, *Gal-9* can promote or inhibit T cell differentiation in the mammalian thymus; however, studies on teleost thymus are limited [9,31,38]. Since the teleost thymus also functions similarly to that of mammals, further studies should evaluate the role of *Gal-9* expression in the thymus.

In previous studies on teleosts, *Gal-9* mRNA expression levels were significantly upregulated in immune-related tissues after bacterial infection [19,20,21,22]. In addition, *Gal-9* mRNA in *Labeo rohita* was upregulated in muscle and heart, which is thought to be an immunologically important function of *Gal-9*, requiring it to be expressed in various tissues throughout the body in response to bacterial infection [21]. *Gal-9* stimulates neutrophils during bacterial infections and contributes to the removal of bacteria, but in mouse model studies, *Gal-9* exacerbates the inflammatory response during sepsis caused by pulmonary *Francisella novicida* infection [39,40]. These findings suggest the need for *Gal-9* in vivo functional analysis during different stages of illness in fish models, especially those of pathogen infection. In rainbow trout, *Gal-9* was upregulated in leukocytes during viral hemorrhagic septicemia virus infection [17], and our results showed that it was also upregulated in the kidney and liver. However, the expression level of *Gal-9* mRNA was significantly downregulated after artificial infection in the spleen and gills. After viral infection with megalocytivirus and RSIV, a lot of the virus was found to accumulate in the gills and spleen, which could be associated with the survival rate [41,42,43,44]. Downregulation of *Gal* expression due to viral infection has been reported in previous studies [45,46,47], and hence, it may be considered an indicator of interference between viral infection and immunomodulation. RSIV inclusion bodies are mainly found in the gills and spleens of infected fish [44,48]. Moreover, the gills and spleen of *Pagrus major* are believed to have decreased *PmGal-9* mRNA expression due to the mobilization or activity of *PmGal-9* protein in the immune system. The functional studies of *Gal-9* against viral diseases in teleosts are still very limited, and further research is needed. These additional studies will be important to properly and effectively control and treat disease by understanding pathogen-derived diseases and the immune system.

## 5. Conclusions

We confirmed the sequence of *PmGal-9* from red sea bream and characterized its deduced amino acid sequence and conserved sequences. Expression profiling of *PmGal-9* mRNA confirmed its expression characteristics and significant changes in expression immune-related tissues in response to pathogen infection.

## Figures and Tables

**Table 1:** Similarities between deduced amino acid sequences of *PmGal-9* and those of homologs in other species.

Common Name	Species	Protein Sequence Length	GenBank Accession No.	Protein Similarity (%)
Rock bream	*Oplegnathus fasciatus*	347	ANN46244	85.6
Gilthead sea bream	*Sparus aurata*	361	XP_030261557	85.0
Large yellow croaker	*Larimichthys crocea*	343	XP_010754381	79.0
Nile tilapia	*Oreochromis niloticus*	324	XP_003458375	72.9
Rainbow trout	*Oncorhynchus mykiss*	341	ACO08221	65.0
Atlantic salmon	*Salmo salar*	344	ACI67584	64.1
Pig	*Sus scrofa*	355	NP_999097	45.4
Cattle	*Bos taurus*	355	NP_001034266	44.0
Human	*Homo sapiens*	355	CAB93851	43.4

## Data Availability

The data presented in this study are available on request from the corresponding author.
